# Investigation of a New Electrode Array Technology for a Central Auditory Prosthesis

**DOI:** 10.1371/journal.pone.0082148

**Published:** 2013-12-02

**Authors:** Roger Calixto, Behrouz Salamat, Thilo Rode, Tanja Hartmann, Bart Volckaerts, Patrick Ruther, Thomas Lenarz, Hubert H. Lim

**Affiliations:** 1 Institute of Audioneurotechnology and Department of Experimental Otology, Hannover Medical University, Hannover, Germany; 2 Cochlear GmbH, Hannover, Germany; 3 Department of Microsystems Engineering (IMTEK) at the University of Freiburg, Freiburg, Germany; 4 Department of Biomedical Engineering, University of Minnesota, Minneapolis, Minnesota; University of Salamanca- Institute for Neuroscience of Castille and Leon and Medical School, Spain

## Abstract

Ongoing clinical studies on patients recently implanted with the auditory midbrain implant (AMI) into the inferior colliculus (IC) for hearing restoration have shown that these patients do not achieve performance levels comparable to cochlear implant patients. The AMI consists of a single-shank array (20 electrodes) for stimulation along the tonotopic axis of the IC. Recent findings suggest that one major limitation in AMI performance is the inability to sufficiently activate neurons across the three-dimensional (3-D) IC. Unfortunately, there are no currently available 3-D array technologies that can be used for clinical applications. More recently, there has been a new initiative by the European Commission to fund and develop 3-D chronic electrode arrays for science and clinical applications through the NeuroProbes project that can overcome the bulkiness and limited 3-D configurations of currently available array technologies. As part of the NeuroProbes initiative, we investigated whether their new array technology could be potentially used for future AMI patients. Since the NeuroProbes technology had not yet been tested for electrical stimulation in an in vivo animal preparation, we performed experiments in ketamine-anesthetized guinea pigs in which we inserted and stimulated a NeuroProbes array within the IC and recorded the corresponding neural activation within the auditory cortex. We used 2-D arrays for this initial feasibility study since they were already available and were sufficient to access the IC and also demonstrate effective activation of the central auditory system. Based on these encouraging results and the ability to develop customized 3-D arrays with the NeuroProbes technology, we can further investigate different stimulation patterns across the ICC to improve AMI performance.

## Introduction

Cochlear implants (CIs) are the most successful neuroprostheses to date with over 200,000 subjects implanted worldwide [1]–[3]. However, in cases where the cochlea or auditory nerve is congenitally malformed or damaged, a CI is not a viable option. In such cases the only commercially available alternative is the auditory brainstem implant [4], [5], a device that stimulates the brainstem with surface electrodes. In general, these patients do not achieve hearing performance levels comparable to CI patients [6].

As an alternative, the auditory midbrain implant (AMI), which targets the central nucleus of the inferior colliculus (ICC) with a penetrating electrode array, was developed [7]. The AMI is in clinical trials and patients receive daily benefits from their implants [8], [9]. However, as with brainstem implants, the AMI also does not achieve performance levels comparable to a CI. Based on recent human and animal studies [9]–[13], one major limitation is that the current single-shank AMI array (consisting of 20 linearly spaced electrodes) cannot sufficiently activate neurons across the three-dimensional (3-D) IC structure for adequate spectral and temporal coding, which are important features for speech perception [14]–[16]. A single-shank array was implanted into the first AMI patients since no 3-D array technologies were available for clinical application [7] and this single-shank array technology had already been shown to be safe for implantation into the ICC [17], [18].

More recently, the European Commission began the NeuroProbes (NP) project to fund and develop new 3-D, chronic array technologies to address the ongoing and significant need for such technologies for neuroscience investigations and clinical applications [19]. We assisted the NP project by testing their new silicon-based array technology in guinea pig experiments to assess whether the NP technology could be used for ICC stimulation and potentially in future AMI clinical trials.

The advantage of silicon-substrate electrode arrays compared to traditional microwires is that they can be fabricated with numerous sites in precise (with micron resolution) and closely spaced (tens of microns) configurations and integrated with electronics. There are other types of silicon-based array technologies currently available in the neural engineering field. The two major devices are known as the Utah array [20]–[22] and the NeuroNexus array (i.e., Michigan Probe [23]–[26]). Both have been successfully used for recording and stimulation applications in acute and chronic animals. The Utah array has also been used in humans for cortical recordings [27]. The major limitation with these devices is that they typically only span a two-dimensional (2-D) space. The Utah array consists of multiple shanks in a 3-D configuration with only one site at the tip of each shank, resulting in a 2-D pattern of sites. The lengths of the shanks can be altered to record or stimulate in different planes but they cannot fully span the 3-D space. The NeuroNexus array is a planar technology in which multiple shanks consisting of several sites along each shank can be configured in a 2-D pattern. There have been attempts at stacking these 2-D arrays into a 3-D configuration using special adapters (examples shown on NeuroNexus website: http://www.neuronexus.com) or superglue [28], but these solutions have resulted in a loss of precision and alignment between the stacked shanks and/or require a bulky interface, which is not favorable for chronic implementation [19].

The NP array takes advantage of the fabrication processes of both types of technologies described above. However, the key difference is that it uses a modular approach to create custom 3-D arrays. The NP array consists of a slim backbone or interface that allows individual shanks and/or groups of 2-D planar arrays to be precisely inserted into this backbone in a Lego©– like fashion [29], [30]. This backbone is slim and connects to a highly flexible ribbon cable to reduce the bulkiness of the interface. It is also possible to incorporate fluidic channels through this interface connecting to some or all of the inserted shanks for drug delivery and chemical sensing, resulting in a fully integrated system [30], [31]. Several prototype NP arrays have been developed and successfully inserted into cortical regions without breakage, have achieved recording of neural activity, and have maintained biocompatibility within brain tissue [32], [33]. Further details on the fabrication process and various configurations of the NP arrays are provided in [29], [30], [32].

Considering the 3-D capabilities of the NP array and the need for 3-D stimulation within the ICC for improving the AMI, we tested the stimulation effects of the NP array in the ICC of the guinea pig. Since the NP technology had not yet been tested for stimulation in an in vivo preparation, our initial objective was to assess if we could electrically stimulate through the NP sites with sufficient current to activate the central auditory system. The shanks also required long lengths of 10 mm to reach the deep location of the ICC, and thus we assessed if the arrays were stiff and strong enough to be inserted through the tissue without breakage. We used 2-D arrays for this study since they were already available and would be sufficient to achieve our objectives, whereas a 3-D array customized to the ICC would require additional time and costs for development. Demonstrating the feasibility of stimulating deep brain structures with the NP arrays in this study will justify further development of 3-D NP arrays for the ICC that can lead to improved stimulation strategies for the AMI.

## Methods

### Anesthesia and surgery

Detailed methods have been presented previously [11], [34]. Briefly, experiments were performed on three albino guinea pigs (494–630 g; DH; Harlan Laboratories, Horst, Netherlands) that were anesthetized with a ketamine (40 mg/kg) and xylazine (10 mg/kg) mixture with additional supplements to maintain an areflexive state. Atropine sulfate (0.05 mg/kg) was administered subcutaneously to reduce bronchial secretions when necessary. Body temperature was maintained at 38±0.5°C with a water heated blanket, and heart rate and blood oxygen levels were monitored via pulse oximetry. The guinea pigs' care and all experiments were carried out in accordance with the German law for animal protection and were approved by the Landesamtes für Verbraucherschutz und Lebensmittelsicherheit (LAVES, registration number 05/1055).

**Figure 1:**
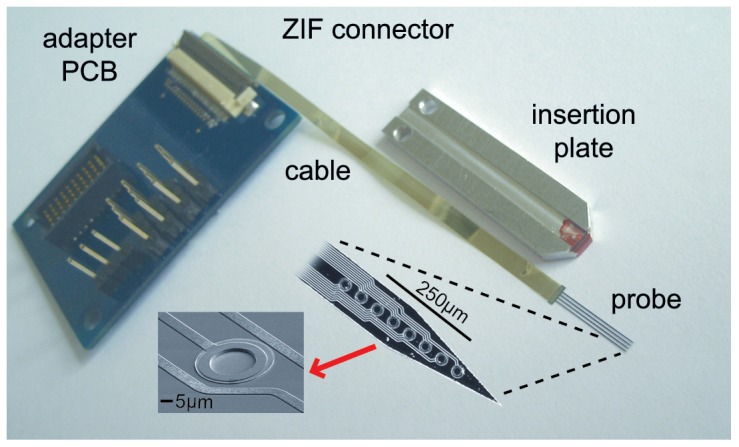
Comb-like, silicon-based NeuroProbes array with four 10-mm-long probe shafts separated by 400 µm. Each shank is comprised of eight IrOx electrode sites. The array is interconnected to a highly flexible polyimide ribbon cable interfacing with a zero insertion force (ZIF) connector on a printed circuit board (PCB) that was connected to the stimulator. For probe insertion, the probe comb is fixed adhesively to the insertion plate and attached to a micromanipulator. The 100-µm-thick probe shanks proved to be stiff enough for insertion into deep brain structures.

The animal was placed in a stereotaxic frame (David Kopf Instruments, Tujunga, CA) with hollow ear bars to allow for calibrated closed-field acoustic stimulation. A craniotomy was performed exposing the right temporal and occipital lobes and the dura was then resected. The occipital lobe was carefully aspirated to provide visual access to the inferior colliculus. The NP array (Fig. 1; 4 shanks, 10 mm long, 8 IrOx sites/shank, 960 µm^2^/site; impedances of 310–630 kΩ at 1 kHz) was then placed at a 45° angle to the sagittal plane and inserted into the inferior colliculus to be aligned along the tonotopic axis of the ICC [35], [36]. Proper array placement in the ICC was confirmed by observing frequency response maps that exhibited an orderly shift from low to high frequencies for superficial to deeper locations, respectively, along a shank [35], [37]. We implanted a 2-D NP array instead of a 3-D version in these initial experiments since 2-D arrays were already available that could achieve the objectives of our study in accessing and acutely stimulating the ICC. Future studies will assess the ability to implant and stimulate a 3-D NP array over longer periods.

For recording the cortical activity, a second array (8 shanks, 200 µm apart, 2 mm long, 4 Ir sites/shank, 413 µm^2^/site; impedances of 1–2 MΩ at 1 kHz; NeuroNexus Technologies, Ann Arbor, MI, USA) was inserted into the primary auditory cortex (A1). The A1 area was identified by its clear tonotopic organization of low to high frequencies with a rostrolateral to caudomedial orientation within the auditory cortex [38], [39]. The array was inserted approximately perpendicular to the cortical surface in an attempt to align each shank along a column in A1 [39], [40]. The depth of the sites was controlled with current source density (CSD) analysis [41]–[43] so that one site per shank was positioned into the main input layer (III/IV) of A1. The one-dimensional CSD approximation provides a consistent representation for the current sinks and sources associated with columnar synaptic activity in the guinea pig auditory cortex [44], [45]. Further details on how to perform the CSD analysis and to identify layer III/IV are provided in [46].

After array placements into ICC and A1, the brain surface was covered with agarose gel to minimize pulsations and drying. The A1 array was used to assess the activation properties of NP stimulation in online and offline analysis.

### Stimulation and recording

Stimulation and recording were performed using a computer interfaced with TDT System 3 hardware (Tucker-Davis Technology, Alachua, FL) using custom written software with Matlab (Mathworks, Natick, MA). Acoustic stimulation was used to guide placement of arrays based on neural response patterns. In each animal, we then identified one stimulation site per shank on the NP array for a total of four sites that were in similar frequency regions of the ICC. We electrically stimulated each site with single biphasic pulses (cathodic-leading, 205 µs/phase) and recorded the corresponding neural activity on an A1 site in a similar frequency region. The following frequency regions were stimulated across animals: 15, 16, and 17 kHz. Stimulation level varied from 20–52 dB (in 2-dB steps relative to 1 µA; 10–398 µA). All stimuli were randomly presented for a total of 20 trials, including 20 spontaneous trials (i.e., no stimulus trials), at a rate of 2/s.

### Analysis

**Figure 2:**
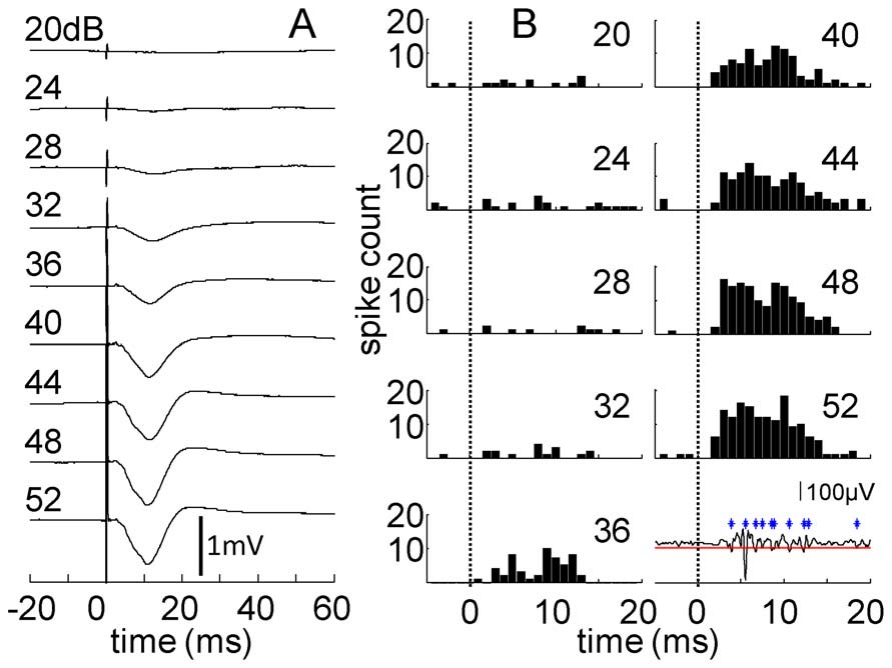
Raw data and PSTH plots. A) Averaged unfiltered raw data (20 sweeps) showing LFP in response to stimulation with an NP site from 20 to 52 dB relative to 1 µA (actual stimuli were 12–52 dB in 2-dB steps). The monotonic increase in LFP size with stimulation amplitude is evident. The LFP threshold is 28 dB in this example. B) PSTHs corresponding to the different stimulation levels indicated in each plot. PSTH bars represent 1 ms bins. The dotted line indicates stimulus onset at 0 ms. Bottom right trace is a single trial filtered for spikes with the artifact removed and showing multi-unit activity in response to a stimulation at 52 dB. Each detected spike is marked by an * with the red line indicating threshold for spike detection. The MUA threshold is 34 dB, which is higher than the LFP threshold.

We analyzed both A1 local field potentials (LFPs) and multi-unit activity (MUA) in response to ICC stimulation. The LFP responses recorded on our main input layer sites generally correspond to the synaptic input into layers III/IV of A1 whereas the MUA corresponds to the spiking pattern of multiple neurons surrounding the recording sites within layer III/IV [42], [47]. LFP analysis was performed on the averaged unfiltered trials after removal of the stimulus artifact (Fig. 2A shows examples with artifact for better visualization of stimulus onset). Artifacts were removed by blanking the 1.5-ms period following stimulus onset for each trial and connecting the points before and after this window with a straight line. We then calculated the magnitude and area of the negative LFP peak as described elsewhere [11], [48]. LFP threshold was defined as the level that elicited a response that was 3.5 times above the average background noise. We used this threshold method because it provided values that were consistent with those determined visually when selecting the level that elicited a noticeable LFP response above the background activity. MUA was displayed as post-stimulus time histograms with 1 ms bins (PSTHs; Fig. 2B) after artifact removal and filtering (300–3000 Hz) of each trial of data and detecting spikes that exceeded three times the standard deviation of the noise floor (Fig. 2B shows example of detected spikes). From the PSTHs, we calculated the driven spike rate (total spikes minus spontaneous spikes within a 30-ms window relative to stimulus onset) across different levels for further analysis. MUA threshold was defined as the lowest level that elicited a visible response above spontaneous activity for two consecutive PSTH level steps.

## Results

We analyzed the LFP and MUA responses recorded in A1 due to ICC stimulation with the NP array. A total of 12 NP sites were stimulated across three animals with various current levels. In all three consecutive animals, we successfully inserted the NP array into the ICC without breakage and could deliver sufficient current through the 12 sites to activate the central auditory system from the ICC up to A1.

### LFP responses

**Figure 3:**
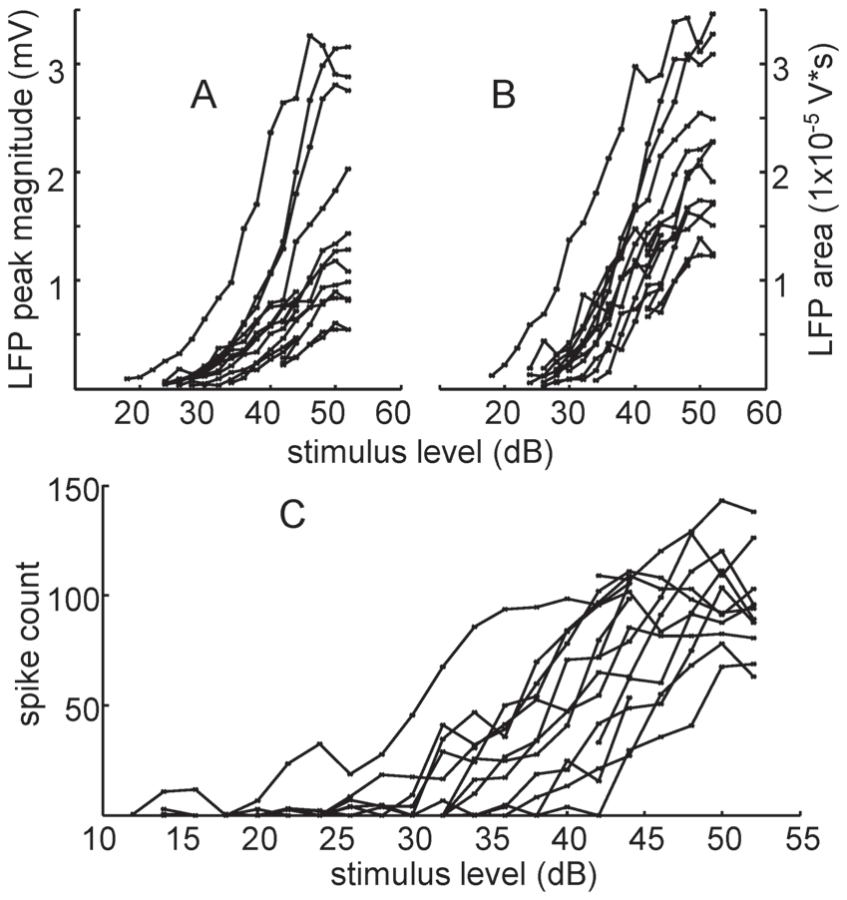
Rate growth curves recorded from A1 and pooled from all 12 stimulated NP sites. A) Growth rate of LFP peak magnitude versus stimulus level (in dB relative to 1 µA). B) Growth rate of LFP area versus stimulus level. C) Growth rate for multi-unit spikes versus stimulus level.

Figure 2A presents A1 LFPs in response to stimulation for varying levels from threshold up to 52 dB (all values in dB relative to 1 µA). The LFP response consisted of a negative deflection that generally exhibited a monotonically increasing magnitude and area with higher current levels (Fig. 3A and 3B, respectively). The mean LFP threshold was 27.7 dB (SD: 5.5), which is approximately 24 µA. Encouragingly, the shape and monotonic nature of these LFP responses appear similar to what has been observed for ICC stimulation with the current AMI array [11], [34]. Monotonic growth functions are important for auditory implants in enabling systematic control over loudness sensations with changes in current level. One main difference is that NP stimulation achieves lower LFP thresholds than what was previously observed for AMI stimulation (∼63 µA; [49]) using similar stimulation and recording parameters (also in guinea pigs). These previous AMI thresholds were obtained by determining the highest level where the evoked potential could not be observed (i.e., a response that was lost in the spontaneous activity), which identifies a threshold level that is lower than what would be selected using our current method (i.e., the lowest amplitude with a detectable LFP). Yet, NP stimulation still achieved lower thresholds suggesting that this difference may be related to the significantly smaller sites of the NP array.

### MUA responses

Figure 2B presents PSTHs for NP stimulation for the same data set used for the LFP responses in Fig. 2A. The MUA responses also generally exhibit monotonically increasing growth functions (Fig. 3C) consistent with the LFP responses. The mean MUA threshold was 33.5 dB (SD: 5.7), which is about 47 µA. Interestingly, these MUA thresholds were higher than what has been observed for AMI stimulation (∼27 µA; [50]) using similar stimulation and recording parameters and the same threshold method as in our study (also in guinea pigs). This result is in contrast to the lower LFP thresholds we observed for NP stimulation compared to AMI stimulation. It is possible that the smaller sites of the NP array will more effectively elicit LFP activity than spike activity (LFP thresholds were significantly lower than MUA thresholds; p<0.0001 using a two-tailed Welch's t-test), whereas the reverse is true for the AMI array. These discrepancies may also be due to the use of different breeds of guinea pigs or different array placements throughout the ICC or A1 across studies.

## Discussion

We were able to successfully insert the NP array into the ICC without breakage. Considering the LFP and MUA thresholds and neural responses presented above, we have also shown that stimulation of the NP array can activate the central auditory system and exhibit similar growth function trends to those previously published for the current AMI array [49], [50]. However, due to the significantly smaller sites of the NP array compared to the current AMI array (960 µm^2^ versus 126,000 µm^2^), one must consider the issue of safety limits for neural stimulation since it depends on the site area.

It is well known that total charge is the main factor eliciting neural activation with central stimulation [51]. Tissue damage to stimulation, on the other hand, is dependent on the synergy of the total injected charge per phase (i.e. sum of charge over time) and charge density per phase (i.e. total charge divided by surface area) [52], [53]. A multi-study comparison has provided a “threshold” for tissue damage that can be modeled with a simple equation, known as the “Shannon curve”, and a safety parameter k (lower k values correspond to more conservative stimulation regimes) that are described in [54]. A k value of 2 has been approximated as the border between safe and unsafe stimulation (for further details, see [53]). However, care should be taken when using this parameter. This equation was largely based on stimulation studies performed with surface electrodes, and thus may overestimate the limits for safe stimulation with penetrating electrodes [55]. Furthermore, the k parameter was calculated based on a limited set of acute stimulation parameters and should be interpreted with care for other stimulation regimes.

**Figure 4:**
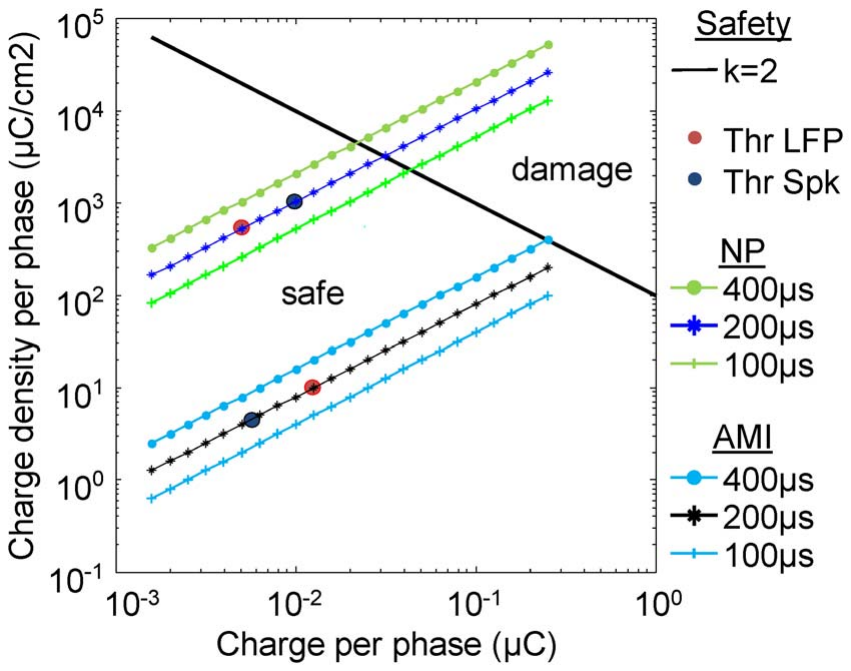
Modeled safe stimulation parameters. Stimulation above the solid black line (k = 2) has been shown to induce tissue damage and co-varies with total charge and charge density per pulse phase. The curves with different symbols reflect how charge density changes with increasing charge per phase for either the NP or AMI sites, i.e., for different site areas, for three different pulse widths each. The local field potential (LFP, red dot, 200 µs/phase) and spike (Spk, blue dot, 200 µs/phase) thresholds obtained from animal studies are labeled on the plot for direct comparison.

Since activation levels (i.e., total charge) on the same order of magnitude were observed for both NP and AMI stimulation, the smaller electrode surface area for the NP sites will lead to higher charge density values, which in turn can lead to greater tissue damage at lower current levels [52], [53]. Figure 4 shows the modeled charge per phase versus charge density per phase for both NP and AMI stimulation using the equation from [54]. Although only pulse widths of 200 µs/phase were delivered experimentally, we have included 100, 200, and 400 µs/phase for comparison. The limits of safe stimulation are approximated by the thick solid line (k = 2). The AMI can be safely stimulated up to about 62 dB (1259 µA) for 200 µs/phase pulses (charge = 0.25 µC and charge density = 199 µC/cm^2^ on the plot). However, NP stimulation can only reach approximately 42 dB (charge = 0.025 µC and charge density = 2623 µC/cm^2^) before exceeding the safety limit. Thus, it is apparent that the total charge range for NP stimulation from threshold to this safety limit (i.e., dynamic range) is smaller than for AMI stimulation.

It is not yet clear if stimulation of smaller and possibly different neural clusters with the NP sites will elicit similar or different auditory percepts as those achieved with AMI sites. This needs to be further investigated in behavioral animal models. However, the key advantage of the NP technology is that a greater number of sites can potentially be implanted throughout the 3-D ICC. It is possible that simultaneous stimulation of multiple sites may enable louder percepts with lower current levels, thus remaining within the safety limits for stimulation. In fact, the ICC normally codes for different sound features through activation of multiple neurons throughout its 3-D structure [56]–[58]. Therefore, a 3-D NP array may provide more realistic activation patterns throughout the ICC to improve overall hearing performance. In this initial feasibility study, we have shown that a 2-D NP array can access and activate central auditory pathways. The next stage of research will be to develop appropriate 3-D arrays that can more fully activate the ICC over longer periods and eventually translate this NP technology into future AMI patients.
